# p53 upregulated modulator of apoptosis sensitizes drug-resistant U251 glioblastoma stem cells to temozolomide through enhanced apoptosis

**DOI:** 10.3892/mmr.2015.3255

**Published:** 2015-01-26

**Authors:** WANG MIAO, XIAODONG LIU, HONGQIN WANG, YIMIN FAN, SHIZHONG LIAN, XIN YANG, XINXING WANG, GENG GUO, QICHAO LI, SIFEI WANG

**Affiliations:** 1 Department of Neurosurgery, The First Hospital of Shanxi Medical University, Taiyuan, Shanxi 030001, P.R. China; 2 Department of Radiotherapy, Shanxi Dayi Hospital, Taiyuan, Shanxi 030032, P.R. China

**Keywords:** p53 up-regulated modulator of apoptosis, glioblastoma, temozolomide, drug resistance, apoptosis, O^6^-methylguanine-DNA-methyltransferase

## Abstract

Malignant glioma is a highly aggressive brain tumor with a poor prognosis. Chemotherapy has been observed to prolong overall survival rate and temozolomide (TMZ), a promising chemotherapeutic agent for treating glioblastoma (GBM), possesses the most effective clinical activity at present, although drug resistance limits its clinical outcome. Growing evidence supports the concept that initial and recurrent GBM may derive from glioblastoma stem cells, which may be responsible for drug resistance. However, the molecular mechanisms underlying this resistance remain to be elucidated. In the present study, a TMZ-resistant GBM cell line, U251R, was developed and subsequently divided into two subpopulations according to the CD133 immunophenotype. No significant difference was identified in the expression of O^6^-methylguanine-DNA-methyltransferase (MGMT) between CD133^+^ U251R cells and CD133^−^ U251R cells, whereas the CD133^+^ cell population was more resistant to TMZ-induced growth inhibition and cell death. TMZ achieves its cytotoxic effect by inducing DNA lesions and p53 upregulated modulator of apoptosis (PUMA) is an essential mediator of DNA damage-induced apoptosis independently of p53 status. Therefore, whether PUMA effectively enhances growth suppression and induces apoptosis when combined with TMZ was investigated. Consequently, it was found that adenoviruses expressing wild-type-PUMA not only lead to the apoptosis of CD133^+^ U251R cells alone, but also significantly increase their sensitivity toward TMZ by elevating the Bcl-2-associated X protein/B-cell lymphoma-2 ratio without alterations in MGMT expression. Therefore, PUMA may be a suitable target for intervention to improve the therapeutic efficacy of TMZ.

## Introduction

Malignant glioma is the most common primary tumor of the brain and the most aggressive human tumor (1,2). Despite intense efforts to find effective treatments, high-grade malignant gliomas, particularly glioblastoma (GBM), are currently incurable. Temozolomide (TMZ), a first-line chemotherapeutic agent, in addition to radiotherapy and surgical resection, improved the overall survival rate and progression-free survival of patients with newly diagnosed GBM (3,4). However, numerous GBM cases gradually develop resistance to TMZ treatment despite the initial benefit. Intrinsic or acquired chemoresistance is a major cause of TMZ treatment failure in GBM patients (5). TMZ is a DNA-alkylating agent that causes DNA damage predominantly by methylating the O^6^-position of guanine, eventually inducing cell death (6). Previous studies have suggested a significant inverse association between cellular ability to repair O-alkyl guanine and sensitivity to methylating agents (7–10). O^6^-methylguanine-DNA methyl-transferase (MGMT) is a DNA repair protein, which restores the structural integrity of O^6^-alkylated guanine bases by direct transfer of the alkyl group to an internal cysteine residue (Cys145) within its active site (5,7). The level of MGMT expression positively correlates with *in vitro* and *in vivo* glioma resistance to TMZ and bis-chloroethylnitrosourea (11,12).

Previously, evidence in certain malignancies has supported the theory that various types of tumor are organized in a hierarchy of heterogeneous cell populations (13,14). The capability to sustain tumor formation and growth is exclusively due to a small proportion of tumor cells termed cancer stem cells or tumor-initiating cells, which are termed glioblastoma stem cells (GSCs) in GBM (15). In addition, a number of studies suggest that GSCs are closely associated with resistance to radiotherapy and chemotherapy although the underlying mechanism remains to be elucidated (16–23).

Resistance to apoptosis is a fundamental part of carcinogenesis and is critical for chemotherapeutic drug resistance (24). It is well established that the p53 pathway is critical in detecting DNA damage and regulating the signaling pathways required to mediate apoptosis. p53 upregulated modulator of apoptosis (PUMA) was identified as a principal mediator of p53-dependent and independent apoptotic pathways (25). PUMA is a B-cell lymphoma 2 (Bcl-2) homology 3 protein and a potent pro-apoptotic Bcl-2 family member (26). A previous study demonstrated that PUMA was able to induce apoptosis of glioma cells and overexpression of PUMA induces activation of caspases and cytochrome c release (27).

It has been the focus of ongoing preclinical and clinical research to understand the mechanisms underlying TMZ resistance in human glioma and develop more effective strategies to overcome chemotherapy resistance (28). This suggested that a reduction of PUMA may be responsible for TMZ resistance in U251R GSCs. Therefore, the present study aimed to examine whether the introduction of PUMA into the TMZ resistant CD133^+^ U251R cells may reverse the drug resistance of U251R GSCs cells in response to TMZ treatment.

## Materials and methods

### Cell culture and treatments

The human glioma cell line, U251MG, with partial TMZ sensitivity was purchased from the Chinese Academy of Sciences Cell Bank (Shanghai, China). U251MG cells were cultured in the following complete medium: Dulbecco’s modified Eagle’s medium (DMEM; Invitrogen Life Technologies, Carlsbad, CA, USA), 10 mM HEPES (Invitrogen Life Technologies), 10% heat-inactivated fetal bovine serum (Irvine Scientific, Santa Ana, CA, USA), 100 U/ml penicillin and 100 *μ*g/ml streptomycin. Over a period of 6 months, TMZ-resistant U251MG cells, termed U251R, were obtained by sequentially increasing the concentration of TMZ. Subsequently, U251R cells were divided into two subgroups, CD133^+^ U251R cells and CD133^−^ U251R cells using positive magnetic activated cell separation. The medium was replaced with stem cell-permissive medium containing DMEM/F-12 with high glucose, 20 ng/ml basic fibroblast growth factor (Peprotech, Rocky Hill, NJ, USA), 20 ng/ml of epidermal growth factor (Peprotech), 4 *μ*g/ml B27 supplement (Gibco-BRL, Carlsbad, CA, USA), 0.1 mg/ml Glutamax (Invitrogen Life Technologies), 100 U/ml penicillin G and 100 *μ*g/ml streptomycin (Gibco-BRL). In all experiments, cells were maintained at 37°C in a humidified atmosphere with 5% CO_2_. The cells were observed and photoed with an Olympus IX71 inverted fluorescence microscope (Olympus, Tokyo, Japan). TMZ was obtained from Schering Corporation (Kenilworth, NJ, USA) and dissolved in dimethyl sulfoxide (DMSO; Sigma-Aldrich, St. Louis, MO, USA) to produce a 150 mmol/l stock solution. The stock solution was diluted to appropriate concentrations with cell culture media (DMSO≤0.3%, v/v). For combination treatments, cells were infected with adenoviruses for 16–18 h prior to TMZ treatment.

### Recombinant adenoviruses

Adenoviruses expressing wild-type PUMA (Ad-PUMA) bound to DsRed were constructed using the Ad-Easy system from Qbiogene (Irvine Scientific). High-titer viruses were produced in HEK-293 cells and purified by a series of CsCl gradient ultracentrifugation, as previously described (29). The cells were infected with Ad-DsRed-PUMA, diluted in cell culture medium in the absence of serum or antibiotics at the indicated multiplicity of infection (MOI) for 2 h at 37°C.

### Magnetic cell sorting and flow cytometry

U251R cells were dissociated and resuspended in phosphate-buffered saline (PBS) containing 0.5% bovine serum albumin and 2 mmol/l EDTA. Subsequently, U251R cells were labeled with 1 *μ*l CD133/l microbeads per 1 million cells using the CD133 Cell Isolation kit (MACS; Miltenyi Biotec, Bergisch-Gladbach, Germany). Following separation, CD133^+^ and CD133^−^ cells were seeded into the same stem cell-permissive medium. The efficiency of magnetic separation was evaluated by flow cytometry with a fluorescence-activated cell sorting (FACS) Calibur machine (BD Biosciences, Franklin Lakes, NJ, USA) when the cells were incubated at 4°C for an additional 30 min with 10 *μ*l phycoerythrin (PE)-conjugated anti-CD133/2 (Miltenyi Biotec).

### MTT assay

CD133^+^ neurospheres or adherent non-sphere forming cells were prepared into single cell suspension and seeded in 96-well plates at 1,000 cells/well in 200 *μ*l stem cell-permissive medium. MTT (20 *μ*l) solution (final concentration: 0.5 mg/ml; Sigma-Aldrich) was added at 0, 24, 48, 72 and 96 h following substitution of the medium in the presence or absence of TMZ. Subsequently, the cells were incubated for 4 h and the medium was replaced with 150 *μ*l DMSO. The plates were agitated for 15 min and the optical density of the solution in the wells was measured at 490 nm on a Versamax microplate reader (Bio-Rad, Hercules, CA, USA).

### RNA isolation and reverse transcription polymerase chain reaction (RT-PCR)

For RNA isolation, Invisorb Spin Tissue RNA Mini kit 250 (Invitek, Berlin, Germany) was used according to the manufacturer’s instructions. The quantity and purity of the RNA was estimated spectrophotometrically (UV-2450; Shimadzu Corp., Kyoto, Japan) and the integrity was assessed by electrophoresis on a 1% agarose gel. RNA was reverse transcribed using the Omniscript RT kit (Qiagen, Hilden, Germany). The following primers were used: MGMT, forward 5′-GTGAAATGAAACGCACCACAC-3′ and reverse 5′-GGAACTCTTCGATAGCCTCGG-3′; Glyceraldehyde-3-phosphate dehydrogenase (GAPDH) gene (served as an internal control), forward 5′-ACACCCACTCCTCCACCTTT-3′ and reverse 5′-TAGCCAAATTCGTTGTCATACC-3′. The amplicon size for GAPDH was 225 bp and MGMT was 120 bp. The PCR conditions for MGMT genes were 29 cycles at 94°C for 45 sec, 54°C for 30 sec followed by 72°C for 45 sec. The PCR conditions for GAPDH were 30 cycles at 94°C for 30 sec, 54°C for 30 sec followed by 72°C for 1 min. Amplified PCR products were electrophoresed on a 1.5% (w/v) agarose gel (Sigma-Aldrich) containing 0.5 *μ*g/ml ethidium bromide. Relative levels of the target genes were quantified using a computer-aided imaging analysis system, AlphaEaseFC™ (Alpha Innotech Corp., San Leandro, CA, USA).

### Western blot analysis

Cultured cells were washed with PBS and protein was extracted using a lysis buffer (50 mM HEPES pH 8.0, 50 mM NaCl, 1% Triton X-100, 10% glycerol, 1 mM MgCl_2_, 1.5 mM EDTA, 20 mM β-glycerophosphate, 50 mM NaF, 1 mM Na3VO4, 10 *μ*g/ml aprotinin, 1 *μ*M pepstatin A, 1 mM phenylmethylsulphonyl fluoride). The protein concentrations were determined using a DC protein assay kit (Bio-Rad). Whole cell lysates containing 50 *μ*g of protein were fractionated using 12% SDS-PAGE (Bio-Rad) and transferred onto a nitrocellulose membrane (Bio-Rad). The membrane was blocked with Tris-buffered saline plus 0.1% Tween 20 (Sigma-Aldrich) containing 5% non-fat milk for 1 h at room temperature followed by incubation overnight with the following primary antibodies: Mouse anti-human monoclonal anti-MGMT (1:500), rabbit anti-human monoclonal anti-PUMA (1:500), rabbit anti-human polyclonal anti-Bcl-2 (1:1,000), rabbit anti-human polyclonal anti-Bax (1:1,000) and mouse anti-human monoclonal anti-GAPDH, which were all purchased from Santa Cruz Biotechnology, Inc. (Santa Cruz, CA, USA) at 4°C. The membrane was washed three times and incubated with horseradish peroxidase-conjugated monoclonal goat anti-rabbit immunoglobulin G (1:1,000) or rabbit anti-mouse (1:1,000) secondary antibodies (Cell Signaling Technology, Inc., Beverly, MA, USA) for 1 h. Immunoreactive protein was detected using chemiluminescence with Kodak X-AR film (Eastman Kodak, Rochester, NY, USA).

### Apoptosis assay

Trypsin was purchased from Sigma-Aldrich and the concentration of trypsin was 0.25%. The cells were digested with 0.25% trypsin for 3–5 min at 37°C. Following trypsin enzyme digestion, adherent and floating cells were harvested and washed twice in ice-cold PBS. Subsequently, cells were resuspended at a density of 1×10^6^ cells/ml in 1X binding buffer (Sigma-Aldrich) followed by the addition of 5 *μ*l of Annexin V-fluorescein isothiocyanate and 5 *μ*l of propidium iodide (Sigma-Aldrich). Following this, cells were incubated in the dark at room temperature for 15 min, cell death was determined using a flow cytometer (FACSCalibur; BD Biosciences). Data were analyzed using CellQuest Pro software, version 6 (BD Biosciences).

### Tumor xenografts

The present study was approved by the ethics committee of the Institutional Animal Care and Use Committee of Shanxi Medical University (Taiyuan, China) and all procedures were conducted in accordance with the criteria outlined in the international guidelines for the care and treatment of laboratory animals. A total of 24, 4-week-old female BALB/C-nude mice weighing 18–20 g were purchased from Hunan Silaikejingda Laboratory Animal Technology Co., Ltd. (Changsha, China) and housed in pathogen-free conditions. The mice were subjected to subcutaneous inoculation into the bilateral axillae with a total of 4×10^6^ (200 *μ*l) U251R cells per mouse. Treatment was initiated when the tumors reached a mean volume of 30–40 mm^3^. The nude mice were randomly divided into the following four groups (n=6): i) PBS control; ii) TMZ; iii) Ad-PUMA and iv) TMZ+Ad-PUMA. A total of 50 *μ*l PBS alone or Ad-PUMA (5×10^8^ PFU/ml) in 50 *μ*l PBS was administered by direct injection into the tumor and TMZ (150 mg/m^2^/day) was administered by gastric catheter. Subsequently, treatment was administered once every 3 days and was provided five times in total. Tumor growth was monitored every other day. Tumor volume (V) was calculated as (LxW^2^)/2, where L=length (mm) and W=width (mm) as described previously (30). On day 40, animals were euthanized and tumors were excised and weighed. The tumor specimens of nude mice were fixed in formalin, embedded in paraffin and cut 4.0 *μ*m in thickness. Sections were stained with hematoxylin and eosin and subjected to terminal deoxynucleotidyl transferase biotin-dUTP nick end labeling (TUNEL) staining for detection of apoptotic cells using the TUNEL apoptotic detection kit (Upstate Biotechnology, Inc., Lake Placid, NY, USA) according to the manufacturer’s instructions.

### Statistical analysis

All experiments were repeated at least three times. The data are expressed as the mean ± standard deviation. Statistical analysis was performed using IBM SPSS Statistics 19.0 software (SPSS, Inc., Chicago, IL, USA). Student’s t test or one-way analysis of variance were performed to determine significant differences. P<0.05 was considered to indicate a statistically significant difference.

## Results

### Drug-resistant GBM cell line, U251R may be subclassified according to its CD133 immunophenotype

**Figure 1:**
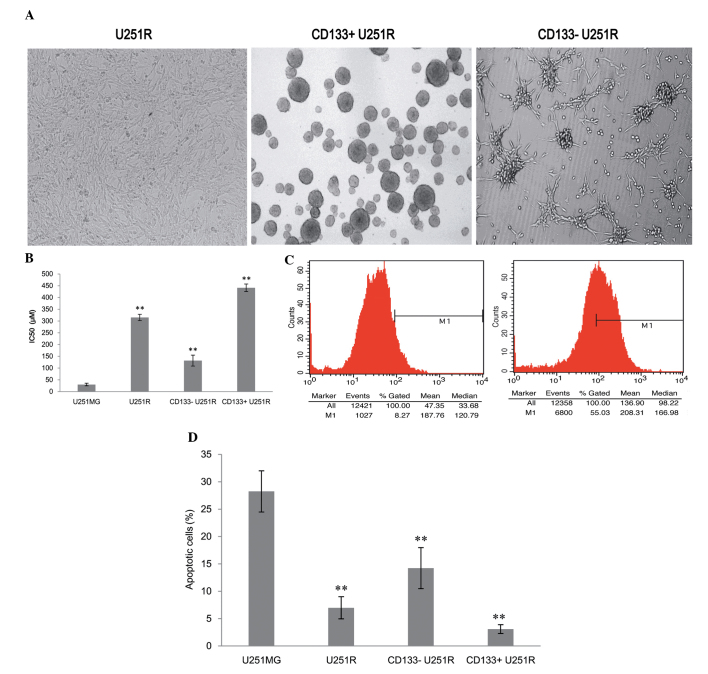
Establishment of a TMZ-resistant glioblastoma cell line. (A) TMZ-resistant glioblastoma cell line U251R, which was established via progressive increases in TMZ concentration for 6 months, can be subclassified into CD133^+/−^ U251R subpopulations according to the CD133 immunophenotype. (B) Cells were treated with different doses of TMZ for 24 h. The inhibition rate of U251MG, U251R, CD133^+/−^ U251R cells was detected using an MTT assay following treatment with different doses of TMZ and the half maximal inhibitory concentration (IC50) was calculated. (C) Percentage of CD133 positive cells in U251MG and U251R cells examined using fluorescence-activated cell sorting. Left: U251MG. Right: U251R. (D) Annexin-V/propidium iodide double-staining assay was performed to analyze the effect of 200 *μ*mol/l TMZ treatment for 48 h on the apoptotic rates of U251MG, U251R, CD133^+^ U251R cells or CD133^−^ U251R cells. Values are presented as the mean ± standard error of the mean. ^**^P<0.05, compared with the U251MG group. TMZ, temozolomide.

U251MG cells were exposed to TMZ from an initial induction dose of 1.25 *μ*mol/l to a final dose of 100 *μ*mol/l. After 6 months, the stable TMZ-resistant variant was obtained by progressively increasing the concentration of TMZ, termed U251R. Subsequently, the U251R cells were isolated with a magnetic activated cell sorting (MACS) kit based on the CD133 immunophenotype. The cells were divided into two groups, CD133^+^ U251R cells and CD133^−^ U251R cells. The two groups of cells were cultured for ~2–3 days in stem cell media. Floating tumor neurospheres were observed in CD133^+^ U251R cells whereas CD133^−^ U251R cells were attached to the bottom of the wells with no tumor spheres forming (Fig. 1A). Their chemosensitivity was evaluated by MTT assay and U251R cells exhibited apparent resistance to TMZ compared with parent U251MG cells (P<0.05; Fig. 1B).

### CD133^+^ U251R cells are more resistant to TMZ-induced cell growth inhibition and apoptosis

The chemosensitivity of GBM cells to TMZ was evaluated by MTT assay. The results revealed that compared with parental U251MG cells (29.83±5.61 *μ*M), the half maximal inhibitory concentration (IC50) values of TMZ-treated cells exhibited a 10.6-fold increase in U251R cells (315.20±13.38 *μ*M), a 4.4-fold increase in CD133^−^ U251R cells (131.85±23.31 *μ*M) and a 14.8-fold increase in CD133^+^ U251R cells (441.24±16.27*μ*M). This indicated that CD133^+^ U251R cells possessed the strongest drug resistance to TMZ-induced cell growth inhibition (F=739.18; P<0.05; Fig. 1B). This was further confirmed by flow cytometry analysis results demonstrating that the percentage of CD133^+^ U251R cells increased significantly (55.03%) compared with parental cells U251MG (8.27%; P<0.05; Fig. 1C). Therefore, CD133^+^ cells or GSCs were enriched in drug resistant U251R cells.

It is well established that resistance to apoptosis is a hallmark of virtually all types of human cancer, including GBM and is also involved in drug resistance. Therefore, in order to analyze the effects of 200 *μ*mol/l TMZ treatment for 48 h on the apoptotic rates of U251MG, U251R, CD133^+^ U251R cells or CD133^−^ U251R cells, an annexin-V/propidium iodide (PI) double-staining assay was performed. Compared with parent U251MG cells, apoptotic rates were significantly decreased in the other three groups (F=89.535; P<0.05). Apoptotic cells were hardly detectable in CD133^+^ U251R (Fig. 1D).

### No significant differences are observed in MGMT expression levels between CD133^+^ U251R and CD133^−^ U251R cells

**Figure 2:**
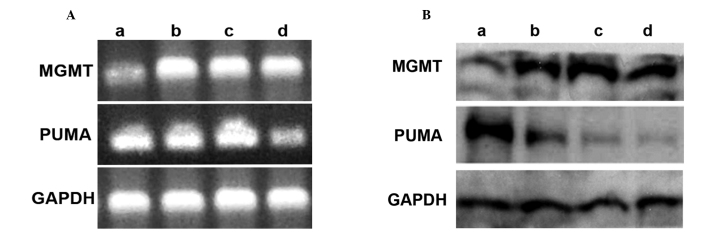
Expression of MGMT and PUMA in U251MG, U251R and CD133^+/−^ U251R cells. (A) Protein expression of MGMT and PUMA in cells of the four groups was analyzed by western blotting. (B) MGMT and PUMA mRNA levels were confirmed by RT-PCR. GAPDH was amplified as an internal control for western blotting and RT-PCR. (a) U251MG; (b) U251R; (c) CD133^−^ U251R; (d) CD133^+^ U251R. MGMT, O^6^-methylguanine-DNA-methyltransferase; PUMA, p53 upregulated modulator of apoptosis; RT-PCR, reverse transcription polymerase chain reaction; GAPDH, glyceraldehyde-3-phosphate dehydrogenase.

Sensitivity of glioma cells to TMZ has been reported to be significantly involved in the level of MGMT repair activity in cells. Thus, the mRNA and protein expression of MGMT was detected using RT-PCR and western blot analysis, respectively. The results demonstrated that the mRNA and protein expression of MGMT in U251MG was significantly lower than that in U251R, CD133^+^ U251R or CD133^−^ U251R cells (P<0.05). However, no significant differences were identified in the remaining three groups (P>0.05; Fig. 2). Considering that CD133^+^ U251R cells are more resistant to TMZ-induced cell growth inhibition and apoptosis, it was hypothesized that there may be additional drug resistance mechanisms in CD133^+^ U251R cells in addition to the mechanism of MGMT-mediated resistance to TMZ.

### Decreased PUMA expression may be in accordance with drug-resistance of GSCs to TMZ

PUMA expression may be induced by p53 or DNA-damaging agents. The expression of PUMA was analyzed and it was observed that the three GBM drug-resistant variant cell lines presented a reduced PUMA expression compared with their parental U251MG cells (P<0.05). Notably, the PUMA protein or mRNA band of the CD133^+^ U251R cell line group exhibited a lower expression level than that of U251R and CD133^−^ U251R cells (P<0.05; Fig. 2). These results suggested that PUMA-mediated apoptotic pathways may be impaired in drug-resistant GSCs. Therefore, loss of PUMA may alter the sensitivity of GBM cells to TMZ treatment.

### Ad-PUMA markedly sensitizes CD133^+^ U251R cells to TMZ through induction of apoptosis

**Figure 3:**
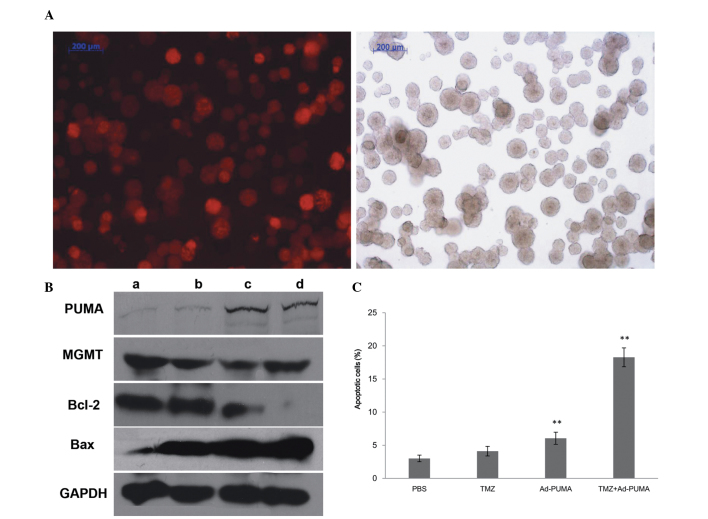
Ad-PUMA sensitizes CD133^+^ U251R cells to TMZ treatment through apoptosis induction. (A) In the left picture, red fluorescence is observed in the CD133^+^ U251R transfected with Ad-Dsred-PUMA. The right picture shows the CD133^+^ U251R cells under normal white light. (B) PUMA, MGMT, Bcl-2, Bax and internal control GAPDH protein expression was analyzed by western blotting. (a) PBS control; (b) 200 *μ*mol/l TMZ treatment for 48 h; (c) 100 multiplicity of infection Ad-PUMA; (d) Ad-PUMA+TMZ. (C) Apoptosis was quantitated by Annexin-V/propidium iodide assay following different treatments. Values are presented as the mean ± standard error of the mean. ^**^P<0.05, compared with the PBS control group. MGMT, O^6^-methylguanine-DNA-methyltransferase; PUMA, p53 upregulated modulator of apoptosis; AD-PUMA, adenoviruses expressing wild-type PUMA; PBS, phosphate-buffered saline; Bcl-2, B-cell lymphoma 2; TMZ, temozolomide; GAPDH, glyceraldehyde-3-phosphate dehydrogenase; Bax, Bcl-2-associated X protein.

To investigate the hypothesis that exogenous expression of PUMA may enhance sensitivity of CD133^+^ U251R cells to TMZ treatment, the recombinant adenovirus Ad-DsRed-PUMA was used to treat the CD133^+^ U251R cells at 100 MOI of the adenovirus. The infection efficacy (74.34%) of Ad-PUMA to CD133^+^ U251R was assayed by FACS and red color was observed in cells infected with Ad-DsRed-PUMA with fluorescence microscopy, which indicated that the adenovirus may be used in the treatment (Fig. 3A). Western blot analysis results confirmed that PUMA expression level was significantly upregulated in Ad-DsRed-PUMA infection groups (P<0.05), while no statistical difference was identified between the PBS control group and TMZ alone group (P>0.05; Fig. 3B). Following Ad-PUMA infection at 100 MOI for 2 h, another MTT assay was performed to investigate the change in IC50 of CD133^+^ U251R cells to TMZ. The results demonstrated that exogenous expression of PUMA decreases the IC50 by 3- to 8-fold compared with the IC50 of TMZ alone.

Furthermore, as shown in Fig. 3C, following TMZ treatment in the presence or absence of Ad-PUMA, cells were harvested and analyzed for levels of apoptotic cells using FACS. The result demonstrated that few apoptotic cells were observed in CD133^+^ U251R cells solely treated with TMZ or PBS. However, the proportion of apoptotic cells was significantly increased in the Ad-PUMA group and the Ad-PUMA in combination with TMZ group (F=329.433; P<0.05).

### PUMA significantly sensitizes GBM cells to TMZ through elevated Bcl-2-associated X protein (Bax)/Bcl-2 ratio in a MGMT-independent manner

In order to further characterize the PUMA-induced apoptosis in TMZ-resistant cells, the changes in three proteins, MGMT, Bcl-2 and Bax, which were involved in the PUMA-induced apoptotic process were examined. As shown in Fig. 3B, it was observed that when TMZ or PBS alone was used to treat cells, the Bax/Bcl-2 ratio did not alter in CD133^+^ U251R cells (P>0.05). However, when TMZ combined with Ad-PUMA or Ad-PUMA alone was used to treat cells, the Bax/Bcl-2 ratio was markedly increased, indicating that PUMA is significantly associated with apoptosis when combined with TMZ treatment (P<0.05). In addition, as shown in Fig. 3B, compared with control cells, the expression level of MGMT was not statistically significant (P>0.05). These data suggested that the chemoresistance of CD133^+^ U251R cells to TMZ did not only result from the deregulation of MGMT. In addition, the ability of PUMA to induce apoptosis in malignant glioma cells may be independent of MGMT. Possible mechanisms through which PUMA induces apoptosis or sensitizes GBM cells to TMZ may be associated with an elevated Bax/Bcl-2 ratio in an MGMT-independent manner.

### Effect of TMZ-based chemotherapy combined with Ad-PUMA therapy on transplanted tumors induced by U251R in vivo

**Figure 4:**
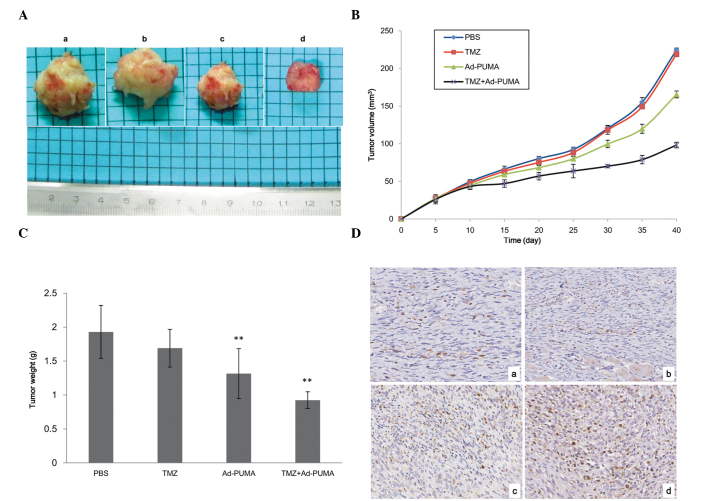
PUMA suppresses the growth of U251R xenograft tumors through induction of apoptosis. (A) Representative images of tumor excised in nude mice 40 days after transplantation (magnification, ×200). (B) Growth curve of tumors treated by four protocols. (C) Average tumor weights of mice 40 days after injection are shown. (D) Apoptosis was analyzed by terminal deoxynucleotidyl transferase biotin-dUTP nick end labeling staining. Brown indicates apoptotic nuclei using the 3,3′-diaminobenzidine substrate. Original magnification, ×200. (Da) PBS control group; (Db) TMZ group; (Dc) Ad-PUMA alone group; (Dd) Ad-PUMA+TMZ group. Values are presented as the mean ± standard error of the mean. ^**^P<0.05, compared with the PBS control group. PUMA, p53 upregulated modulator of apoptosis; AD-PUMA, adenoviruses expressing wild-type PUMA; PBS, phosphate-buffered saline; TMZ, temozolomide.

Following on from *in vitro* experiments, which revealed that Ad-PUMA sensitizes the drug resistant glioma cells to TMZ treatment, it was further investigated whether this sensitization effect may also be detected *in vivo* in tumor xenograft animal models. U251R cells were injected subcutaneously into the bilateral axillae of nude mice and secondary tumors were observed in all injected mice following cell inoculation. Subsequently, tumors initiated by U251R cells were treated with PBS, TMZ alone, Ad-PUMA alone and combined TMZ plus Ad-PUMA, respectively. As shown in Fig. 4A and B, the average tumor volume in the Ad-PUMA+TMZ group and the Ad-PUMA group 40 days after transplantation was smaller than the other two groups (P<0.05). Ad-PUMA combined with TMZ suppressed the growth of subcutaneous tumors more potently than Ad-PUMA alone. Similarly, tumors treated with Ad-PUMA in combination with TMZ were significantly lighter than the remaining three groups (P<0.05; Fig. 4C). In addition, tumor sections were stained using a TUNEL kit to evaluate the rates of apoptosis. The results confirmed that Ad-PUMA may induce apoptosis of xenograft tumors alone by enhanced apoptosis induced by TMZ treatment. By contrast, apoptotic cells were almost undetectable in tumors of PBS control treatment or the TMZ alone group (Fig. 4D). These results suggested that U251R tumors possess significant drug resistance to TMZ and Ad-PUMA may only partly reverse TMZ drug-resistance of tumors initiated by U251R cells to TMZ. Once Ad-PUMA was used to infect the tumors, which were simultaneously treated with TMZ, the tumor volume and weight were markedly decreased, suggesting that the combination of TMZ with Ad-PUMA was effective and its antitumor effect may be due to induction of apoptosis.

## Discussion

The alkylating agent, TMZ is currently the first choice chemotherapy drug for GBM treatment with the best clinical efficacy (31). However, endogenous or acquired resistance to TMZ limits the clinical outcome of GBM patients and is also an important cause of GBM relapse (32). Therefore, in-depth understanding of TMZ resistance mechanisms is key to overcoming TMZ resistance and improving the prognosis of patients with GBM. Through intervening in associated mechanisms, tumor cell sensitivity to TMZ may be restored (33).

With the emergence of the cancer stem cell theory, the aim of therapeutics shifted toward eradicating cancer stem cells (34,35). Significant research efforts have been devoted to understanding and overcoming this critical issue. Therefore, understanding and exploiting the chemoresistance mechanisms of GSCs to TMZ treatment in gliomas is an important strategy for the development of new targeted therapies. In the present study, drug resistant GBM cells, U251R, induced through progressive increases in TMZ treatment for 6 months were developed. Cells were separated into two subpopulations by MACS based on the CD133 immunophenotype and subsequently cultured in stem cell-permissive medium. It was observed that the growth rate of CD133^−^ U251R cells was slower than that of CD133^+^ U251R cells. In addition, it was also noted that the IC50 of CD133^+^ U251R cells was significantly higher than CD133^−^ U251R, U251 MG and U251R cells. FACS analysis suggested that CD133^+^ cells are abundant in U251R cells. Notably, no statistical differences in the expression of MGMT were identified between CD133^+^ U251R and CD133^−^ U251R cells, although the two exhibited higher expression levels than U251 MG cells. This demonstrated that the resistance of CD133^+^ U251R against TMZ is not limited to MGMT expression levels and there may be additional mechanisms. Liu *et al* (22) also found that CD133^+^ cells in GBM were more resistant to TMZ, paclitaxel and VPl6 than the negative cells, which may be induced by the higher expression levels of breakpoint cluster region pseudogene 1 and MGMT, as well as the anti-apoptosis protein and inhibitors of apoptosis protein families in CD133^+^ cells.

Prevention of apoptosis is a hallmark of cancer and confounds the efficacy of cancer chemotherapy (24). TMZ exerts its antitumor effects predominantly through cell death induced by DNA double-strand breaks (6). Whether apoptosis failed in CD133^+/−^ U251R cells following TMZ treatment was investigated. In the current study, when compared with parent U251MG, the apoptotic rate of CD133^+/−^ U251R cells was significantly decreased. In order to illustrate this mechanism, changes in the apoptosis-associated molecule, PUMA in U251MG, U251R, CD133^−^ U251R and CD133^+^ U251R were further examined. Western blot analyses indicated that compared with the parental U251MG cells, PUMA expression levels were significantly decreased in CD133^+/−^ U251R cells, particularly in CD133^+^ U251R cells. This suggested that CD133^+^ U251R cells or GSCs mainly contributed to the resistance to TMZ, at least partly, through deregulated PUMA induction.

Wild type p53 protein is established as a potent tumor suppressor (36). However, p53 mutations are common in diverse types of human cancer, including GBM (37). The mutations not only result in p53 protein loss of wild-type function, in the majority of cases, mutant p53 protein acquires an oncogenic gain of function (38–41). PUMA, a p53 downstream molecule has a pro-apoptotic role independent of p53 status. It may destroy cancer cells through interacting with Bcl-2 family members, even when the p53 pathway is dysfunctional (42). Yu *et al* (25) demonstrated that exogenous expression of PUMA protein resulted in a more rapid and profound apoptosis. Ito *et al* (27) confirmed previous findings and revealed that, regardless of p53 status, PUMA expression was directly associated with tumor cell apoptosis. Overexpression of PUMA may lead to a large number of apoptotic cells, which was also accompanied by mitochondrial damage and caspase activation. In a further study, Yu *et al* (43) combined Ad-PUMA with paclitaxel, fluorouracil, cisplatin and etoposide in the treatment of human lung adenoma A549 cells. Ad-PUMA was demonstrated to increase sensitivity of A549 cells to common chemotherapeutic drugs and radiation (43). Ad-PUMA appears to be selectively toxic to cancer cells and more efficient than p53 in inhibiting the growth of cancer cells. It was therefore hypothesized that exogenous PUMA may cause reversion of U251R cells resistant to TMZ. In our subsequent experiments, the analysis of apoptosis by FACS indicated that there were few or no apoptotic cells in the CD133^+^ U251R cells treated with TMZ, however, introduction of exogenous Ad-PUMA not only inhibited cell growth, but also increased their apoptotic rates. It is worth noting that, when combined with TMZ, Ad-PUMA may induce significant growth inhibition and apoptotic responses. Furthermore, using a nude mouse subcutaneous glioma model, the effect of Ad-PUMA combined with TMZ on glioma growth was observed. Consistent with *in vitro* results, Ad-PUMA and TMZ had a synergistic effect on suppressing tumor growth *in vivo*. These results suggested that Ad-PUMA has prospects for application in GBM for TMZ-based chemotherapy.

Previous studies have demonstrated that TMZ treatment alters the expression of pro-apoptotic Bax and anti-apoptotic Bcl-2 involved in the mitochondrial pathway of apoptosis (44,45). Additionally, the ratio of Bax/Bcl-2 correlated with chemotherapy resistance and shortened overall survival (46). Western blot analysis indicated that the ratio of Bax/Bcl-2 was partially decreased in TMZ-resistant cells. However, once these cells were infected with Ad-PUMA, the ratio of Bax/Bcl-2 was significantly upregulated. This demonstrated that PUMA may induce apoptosis through increasing the ratio of Bax/Bcl-2, as high levels of Bax/Bcl-2 is established as an inhibitor of apoptosis (47,48). In addition, this process had no association with MGMT as MGMT protein expression levels did not change.

Together, the present results demonstrate that CD133^+^ U251R cells were more resistant to TMZ treatment than CD133^−^ U251R cells although no significant differences were observed in MGMT protein expression levels between them, the major mediator of TMZ resistance in GBM. By contrast, it was noted that PUMA expression in CD133^+^ U251R cells, a crucial factor in the apoptosis of human glioma cells induced by antitumor drugs, was significantly lower than the other cell lines investigated. This explained, at least partly, why apoptosis was defective in CD133^+^ U251R cells or GSCs. These results suggest a requirement for new strategies against glioma resistance to TMZ. CD133^+^ U251R cells may be a target for future studies. *In vitro* and *in vivo* data suggested that the combination of Ad-PUMA and TMZ is a promising strategy for treating malignant gliomas resistant to chemotherapy. In addition, given the complexity of the p53 pathophysiological network in apoptosis, senescence, growth arrest and the high mutation rate in GBM, approaches of gene therapy that involve increasing PUMA expression may be an improved approach to reverse TMZ resistance in glioma cells. This may induce glioma cell death regardless of p53 status and possess the same efficacy for inducing apoptosis in CD133^+^ tumor cells.
